# MicroRNA-655-3p functions as a tumor suppressor by regulating ADAM10 and β-catenin pathway in Hepatocellular Carcinoma

**DOI:** 10.1186/s13046-016-0368-1

**Published:** 2016-06-04

**Authors:** Gang Wu, Kunming Zheng, Shuguan Xia, Yawei Wang, Xiangyu Meng, Xiaoming Qin, Ying Cheng

**Affiliations:** Department of General Surgery, The First Affiliated Hospital of China Medical University, Shenyang, 110001 China; Department of Breast and Thyroid Surgery, Huaihe Hospital, Henan University, Kaifeng, 475000 China; Department of Gastric Surgery, Liaoning Cancer Hospital and Institute, Shenyang, 110001 China

**Keywords:** miR-655-3p, Hepatocellular carcinoma, ADAM10, β-catenin pathway, Proliferation, Migration and invasion

## Abstract

**Background:**

Increasing evidence suggests that microRNAs (miRNAs) play critical roles in malignant transformation, tumor progression and metastasis. Aberrant miR-655-3p expression has been associated with several cancers. However, the role and underlying mechanism of miR-655-3p in the development of hepatocellular carcinoma (HCC) remains unclear.

**Methods:**

MiR-655-3p expression was detected by quantitative RT-PCR (qRT-PCR) in human HCC tissues and cell lines. Cell proliferation was investigated using MTT and colony formation assays, and cell migration and invasion abilities were evaluated by transwell assay. ADAM10 protein expression was detected by immunohistochemical assay. The target gene and downstream of miR-655-3p were determined by qRT-PCR, western blot and dual-luciferase reporter assays.

**Results:**

miR-655-3p was significantly down-regulated in HCC tissues and HCC cell lines. Low miR-655-3p expression was negatively related to tumor size, portal vein tumor thrombosis (PVTT) status, TNM stage and metastasis status. In addition, miR-655-3p overexpression and depletion decreased and increased HCC cell proliferation, migration and invasion, respectively. Moreover, ADAM10 was identified as a direct target of miR-655-3p, and miR-655-3p down-regulated E-cadherin protein level and inhibits β-catenin pathway by mediating ADAM10.

**Conclusions:**

MiR-655-3p might functions as a tumor suppressor by directly targeting ADAM10 and indirectly regulating β-catenin pathway in the development of progression of HCC. It may be a novel therapeutic candidate target to in HCC treatment.

## Background

Hepatocellular carcinoma (HCC), accounting for 85-90 % of primary liver cancers, is the fifth most frequent malignancy and the second leading cause of cancer-related death in developing countries [1, 2]. There are many risk factors related to HCC, especially hepatitis B and C viruses and liver cirrhosis [3]. Despite increasing significant advances in surgical techniques and diagnostic methods in recent years, the long-time survival is still unsatisfactory mainly due to the high rate of recurrence, metastasis, and emergence of new primary tumors in post operation [4, 5]. Unfortunately, the mechanisms of high rate of recurrence and metastasis in HCC remain unclear. Therefore, it is urgent to identify the new molecular biomarkers in predicting the aggressive biology of HCC and guiding effective treatment for HCC patients.

MicroRNAs (miRNAs) are 18-24 nucleotides, small, single-stranded, non-coding RNAs that regulate gene expression by directly degrading mRNA or suppressing post-transcriptional protein translation usually by binding to the 3’ untranslated region (3’ -UTR) of the respective target mRNAs [6]. It has been confirmed that ample miRNAs can function as tumor suppressors or oncogenes to play important roles in the initiation, promotion and progression of various cancers [7], and aberrant miRNAs expression might be of potential use as a diagnostic and prognostic biomarker for human cancer including HCC. For example, miR-20, miR-182, miR-494, and miR-125b have been proved to regulate tumor cell growth, migration and invasion in HCC [8–11].

There are several miRNAs encoded in 14q32 locus, including miR-655-3p, miR-127-5p, miR-369-3p, miR-544a. MiRNAs on this locus have been reported associated with a metastatic phenotype in clinical cancer samples [12], and ectopic expression of 14q32-encoded microRNAs can reduce cell-autonomous metastatic properties in vitro and inhibit metastasis development in vivo [13]. MiR-655-3p expression is reduced in several cancers and overexpression of miR-655 act as a tumor suppressor by targeting pituitary tumor-transforming gene-1(PTTG1) in esophageal squamous cell carcinoma metastasis [14]. Previous study has demonstrated that miR-655inhibits epithelial mesenchymal transition (EMT) suppressive miRNA by targeting regulate ZEB1 and TGFBR2 inducing inactivation of the TGF-β signaling pathway [15]. In addition, miR-134/487b/655 cluster is also reported to regulate TGF-β induced EMT and gefitinib resistance by targeting MAGI2 in lung adenocarcinoma cells [16]. However, the expression level of miR-655-3p and its roles in the development of HCC have not yet been reported.

In the current study, we investigated the biological function and molecular mechanism of miR-655-3p in HCC. MiR-655-3p was significantly decreased in HCC clinical specimens and cell lines. Overexpression of miR-655-3p suppressed cell proliferation, migration and invasion of HCC in vitro. Further study showed that miR-655-3p could down-regulate E-cadherin and inhibit β**-catenin** pathway by targeting A Disintegrin and Metalloprotease Domain 10(ADAM10). Our findings elucidated the detailed roles of miR-655-3p in HCC and further contribute to offering the effective therapeutic targets for the treatment of HCC.

## Methods

### Patients and specimens

Primary tumor tissues and their corresponding adjacent non-tumorous liver specimens were obtained from 84 patients who were diagnosed with HCC during hepatic resection in the First Affiliated Hospital of China Medical University between July 2013 and July 2014. None had received preoperative radiotherapy or chemotherapy before surgery. Fresh specimens were snap-frozen and stored in liquid nitrogen tanks immediately after resection. The clinical and pathological parameters are shown in Table 1. This study protocol was approved by the Institutional Ethics Committee of China Medical University, and human tumor tissues for this research were obtained with informed consent.

**Table 1:** miR-655-3p expression and clinicopathological features in hepatocellular carcinoma patients

Characteristics	miR-655-3p expression			*p*-value
	Cases	Low	middle/High	
Age (years)				
≥ 53	43	27(62.8 %)	16(37.2 %)	0.69
< 53	41	24(58.5 %)	17(41.5 %)	
Gender				
Male	71	45(63.4 %)	26(36.6 %)	0.242
Female	13	6(46.2 %)	7(53.8 %)	
HBsAg status				
Positive	62	36(58.1 %)	26(41.9 %)	0.404
Negative	22	15(68.2 %)	7(31.8 %)	
AFP (ng/ml)				
≥ 20	48	30(62.5 %)	18(37.5 %)	0.699
< 20	36	21(58.3 %)	15(41.7 %)	
Liver cirrhosis				
Yes	63	38(60.3 %)	25(49.7 %)	0.897
No	21	13(61.9 %)	8(39.1 %)	
Tumour size (cm)				
< 5	44	22(50 %)	22(50 %)	0.035*
≥ 5	40	29(72.5 %)	11(27.5 %)	
PVTT				
Yes	9	9(100.0 %)	0(0.0 %)	0.028*
No	75	42(56 %)	33(44 %)	
TNM Classification				
I + II	50	24(48 %)	26(52 %)	0.004**
III + IV	34	27(79.4 %)	7(20.6 %)	
Tumour differentiation				
Well	37	21(56.8 %)	16(43.2 %)	0.721
Moderate	34	21(61.8 %)	13(48.2 %)	
Poor	13	9(69.2 %)	4(30.8 %)	
Metastasis				
Yes	33	27(81.8 %)	6(18.2 %)	0.001**
No	51	24(47.1 %)	27(52.9 %)	

Abbreviations: *AFP* alpha-fetoprotein, *HBsAg* hepatitis B surface antigen, *TNM* tumor-node-metastasis, *PVTT* portal vein tumor thrombosis. * p<0.05; ** p<0.01

#### HCC cell lines and cell cultures

Seven HCC cell lines, HepG2, SK-hep1, HCCLM3, Huh7, MHCC-97H, MHCC-97 L, BEL-7402, and one normal liver cell line, LO2, were used in this study. The Huh7, BEL-7402, SK-hep1, HCCLM3 and LO2 human cell lines were obtained from the Institute of Biochemistry and Cell Biology at the Chinese Academy of Sciences (Shanghai, China). The HepG2, MHCC97H and MHCC97L cells were obtained from Chinese Academy of Medical Sciences (Beijing, China). HCCLM3, Huh7, HepG2, MHCC97H, MHCC97L were cultured in DMEM medium. BEL-7402, SK-Hep1, LO2 were cultured in RPMI 1640 medium. All the medium was added with 10 % fetal bovine serum (FBS) and 100 units/ml of penicillin and streptomycin (Hyclone, USA). All cells were grown in a humidified incubator with 5 % CO2 at 37 °C.

#### RNA preparation and quantitative real-time PCR

Total RNA from HCC tissue samples and adjacent non-tumorous tissue samples was extracted using Trizol Reagent (Invitrogen, USA) according to the manufacturer’s instructions. To determine mature miRNA expression levels, qRT-PCR was performed using a SYBR Premix Ex Taq (TaKaRa, Japan) on a Thermal Cycler Dice Real Time System (TaKaRa) with the following protocol: 30s at 95 °C followed by two-step PCR for 40 cycles of 95 °C for 5 s and 60 °C for 60s. MiRNA expression levels were normalized against the endogenous U6 small nuclear RNA (U6 snRNA) control. ADAM10 expression was measured by SYBR green qPCR assay and GAPDH was used as an endogenous control. The relative expression level of miR-655-3p in each paired tumor and adjacent non-tumorous tissue was calculated by the 2^-ΔΔCT^ method. The sequences of the PCR primers were as follows: miR-655-3p forward, 5’-CCGCGATAATACATGGTTAACCTC-3’, and reverse primer was Uni-miR qPCR primer (TaKaRa); U6 forward, 5’-CTCGCTTCGGCAGCACA-3’ and U6 reverse, 5’-AACGCTTCACGAATTTGCGT-3’; ADAM10 forward, 5’-CTGCCCAGCATCTGACCCTAA-3’ and reverse, 5’-TTGCCATCAGAACTGGCACAC-3’; GAPDH forward, 5’CTCCTCCTGTTCGACAGTCAGC-3’, and reverse 5’-CCCAATACGACCAAATCCGTT-3’.

#### Oligonucleotides transfection

The miR-655-3p agomiR (agomiR-655-3p), antagomiR (anti-miR-655-3p), small interfering RNA for ADAM10 (siADAM10) and their negative control (Neg.Cont) Oligonucleotides used in this study were purchased from Shanghai GenePharma Co. Ltd. Transfection was performed using Lipofectamine 2000 (Invitrogen) according to the manufacturer’s protocol. The sequences of Oligonucleotides were as follows: agomiR-655-3p, sense 5’-AUAAUACAUGGUUAACCUCUUU-3’ and antisense 5’-AGAGGUUAACCAUGUAUUAUUU-3’; miRNA negative controls, sense 5’-UUCUCCGAACGUGUCACGUTT-3’ and antisense 5’-ACGUGACACGUUCGGAGAATT-3’; anti-miR-655-3p, 5’-AAAGAGGUUAACCAUGUAUUAU-3’; negative control, 5’-UUGUACUACACAAAAGUACUG-3’; Si-ADAM10, 5’-CAGUGUGCAUUCAAGUCAA-3’.

#### Luciferase assay

The wild-type ADAM10-3’UTR(WT) and mutant ADAM10-3’UTR(MUT) containing the putative binding site of miR-655-3p were established and cloned in the Firefly luciferase expressing vector pMIR-REPORT (Obio Technology, China). Liver cancer cells were seeded into 24-well plates the day before transfection, and transfected with either the pMIR-REPORT-ADAM10-3’ UTR-WT or the pMIR-REPORT-ADAM10-3’ UTR-MUT reporter vector, together with the Renilla luciferase-expressing vector pRL-TK (Promega) and agomiR-655-3p or miR-Neg.Cont using Lipofectamine 2000 (Invitrogen). After 48 h, cells were harvested, and firefly and Renilla luciferase activities were measured using the dual-luciferase reporter assay system (Promega, Madison, WI).

#### Cell migration and invasion assays

After 48 h of transfection, cell concentration in each group was adjusted to 2 × 10^5^ cells/mL with serum-free medium. The upper chamber of Transwell chamber (Costar; 24-well insert, pore size: 8 μm) was filled with 200 μl cell suspension, and the lower chamber was filled with 500 μL of medium supplementing 15 % FBS. For the invasion assay, polycarbonate filters coated with 50 μL Matrigel (1:9, BD Bioscience) were placed in a Transwell chamber. Three wells were used for each group. Cells were incubated for 24 h for the migration assay and 48 h for the invasion assay. Then, the cells on the upper surface were wiped slightly using cotton swabs, and the cells on the lower surface were fixed with 4 % paraformaldehyde and stained with 0.1 % crystal violet. The migratory cells were visualized and counted in five random visual fields per insert under an inverted microscope at 200× magnification (Nikon Microphot-FX, Japan).

#### MTT assay

After transfection, 5000 cells/well were seeded in 96-well plates in media containing 10 % FBS and incubated for 0, 24 h, 48 h, 72 h. On the indicated days, 3-(4,5)-dimethylthiahiazo(-z-y1)-3,5-di-phenytetrazoliumromide (MTT) (KyeGEN BioTECH, Nanjing, China) was added into each well according to the manufacturer’s instructions, and the cells were incubated for 4 h at 37 °C. The supernatants were then removed and 150uL of DMSO (Sigma-Aldrich, Germany) was added to per well to dissolve the formazan crystals. Absorbance levels were measured at the wavelength of 490 nm using an automatic microplate reader (Gene, HK). The data derived from triplicate samples are presented as mean ± s.d.

#### Colony formation assay

After transfection, 500 cells per well were counted and seeded in 6-well plates. The plates were incubated for 10 days, then the cells were fixed by 4 % paraformaldehyde and stained using 0.1 % crystal violet. Colonies were counted only if they included 50 cells at least. Triplicate independent experiments were performed and all the visible colonies were calculated manually.

#### Western blot

Cell samples were washed with ice-cold PBS and then lysed by RIPA (Beyotime, China) containing protease inhibitors (Beyotime, China). Cell protein lysates were separated in 10 % SDS-PAGE and then transferred onto a polyvinylidene difluoride (PVDF) membrane (Millipore, USA). The membranes were blocked by 5 % skim milk soluted in TBST buffers, and were incubated with primary antibodies for ADAM10, c-myc (Abcam, UK), E-cadherin, MMP9 (Santa Cruz Biotechnology, USA), β-catenin and cyclinD1 (ProteinTech Group, USA) overnight at 4 °C. PVDF membranes were washed in TBST and incubated with horseradish peroxidase-conjugated secondary antibodies (ProteinTech Group, USA). Antibody against GAPDH (Cell Signaling Technology, USA) was used as an internal control. Antibody against Histone H3 (Abcam, UK) was used as an internal control for nuclear β-catenin. The protein of interest was visualized using ECL Western blotting substrate (Pierce, USA).

### Immunohistochemical (IHC)

Formalin-stabilised liver tissue specimens were embedded in paraffin and cut into 4 μm sections for use in immunohistochemistry. After general deparaffinization, antigen retrieval was carried out for 30 sec with an autoclave using 0.01 mol/l sodium citrate buffer, pH 6.0. H2O2 (0.3 %) was used to block endogenous peroxidase activity for 30 min at 37 °C, and non-specific immunoglobulin binding sites were blocked by normal goat serum for 30 min at 37 °C. Sections were then incubated overnight with primary antibody(ADAM10,1:300, Abcam) at 4 °C, rinsed with PBS, and incubated with the appropriate secondary antibody for 30 min. The peroxidase reaction was developed with 3, 3-diaminobenzidine tetrahydrochloride (DAB).Sections were counterstained with Mayer’s hematoxylin, dehydrated, cleared in xylene, and mounted in Permount.

#### Statistical analysis

The statistical analyses were performed using the SPSS 17.0 software. Parametric data were presented as mean ± SEM, and differences between each group were analyzed using the Student’s t-test. The association between miR-655-3p relative expression and the clinicopathological parameters was evaluated by the χ2 test or Fisher’s exact test when appropriate. All of the p-values reported were two-sided, and significance was defined as *p* < 0.05.

## Result Analysis

### MiR-655-3p expression in HCC tissues and cell lines

**Fig. 1:**
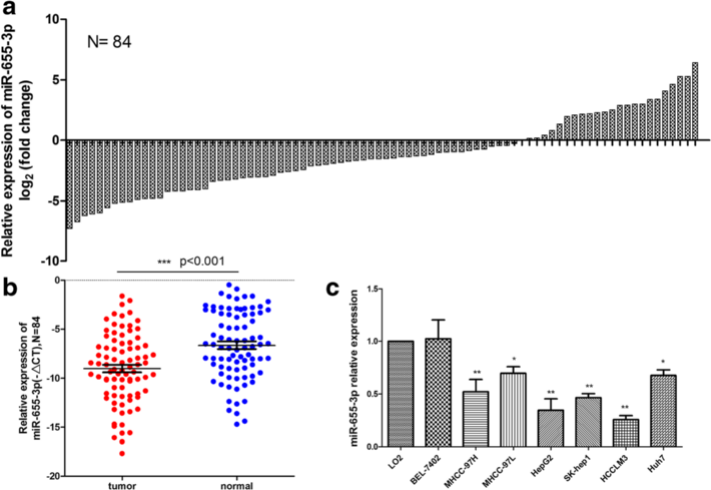
MiR-655-3p is low-expressed in HCC tissues and cell lines. **a** QRT-PCR analysis of miR-655-3p expression in 84 pairs HCC and their corresponding adjacent nontumorous livers tissues. The expression of miRNA was normalized to U6 snRNA. **b** Relative miR-655-3p expression levels in HCC tissues and adjacent normal regions; **c** QRT-PCR analysis of miR-655-3p expression in HCC cells (Bel-7402, MHCC-97 L, MHCC-97H, HepG2, SK-Hep1, HCCLM3, Huh7) and normal hepatocytes (LO-2)

To analysis the miRNA-655-3p expression pattern, 84 pairs of HCC tissues and adjacent non-tumorous liver tissues were detected by qRT-PCR. The result showed that down-regulation of miR-655-3p was observed in 61 (72.6 %) cases of HCC tissues, which was significantly lower than that in matched non-tumorous tissues (*P* <0.001, Fig. 1a, b). In cell level, miR-655-3p expression was lower in the HCCLM3, HepG2, SK-hep1, MHCC-97H, Huh7, MHCC-97 L cell lines than that in the normal liver cell line LO2 (Fig. 1c). All the above results indicated that miR-655-3p was down-regulated in HCC.

### Association of miR-655-3p expression with clinicopathological features

In order to explore the potential clinical significance of miR-655-3p in HCC patients, the cases were divided into miR-655-3p low-expression group (*n* = 51) and mid/high-expression group (*n* = 33), according to the relative ratio of miR-655-3p expression in tumor/adjacent non-tumor < or > 0.5. The correlation between miR-655-3p expression and clinicopathological characteristics was shown in Table 1. MiR-655-3p expression was positively associated with tumor size (*p* = 0.035), PVTT (*p* = 0.028), TNM stage (*p* = 0.004) and metastasis (*p* = 0.001), respectively. However, it was no correlations with gender, age, preoperative serum AFP and histological differentiation. Based on these findings, we speculated miR-655-3p might play a vital role in HCC development.

### Ectopic expression of miR-655-3p inhibits HCC cell lines proliferation

**Fig. 2:**
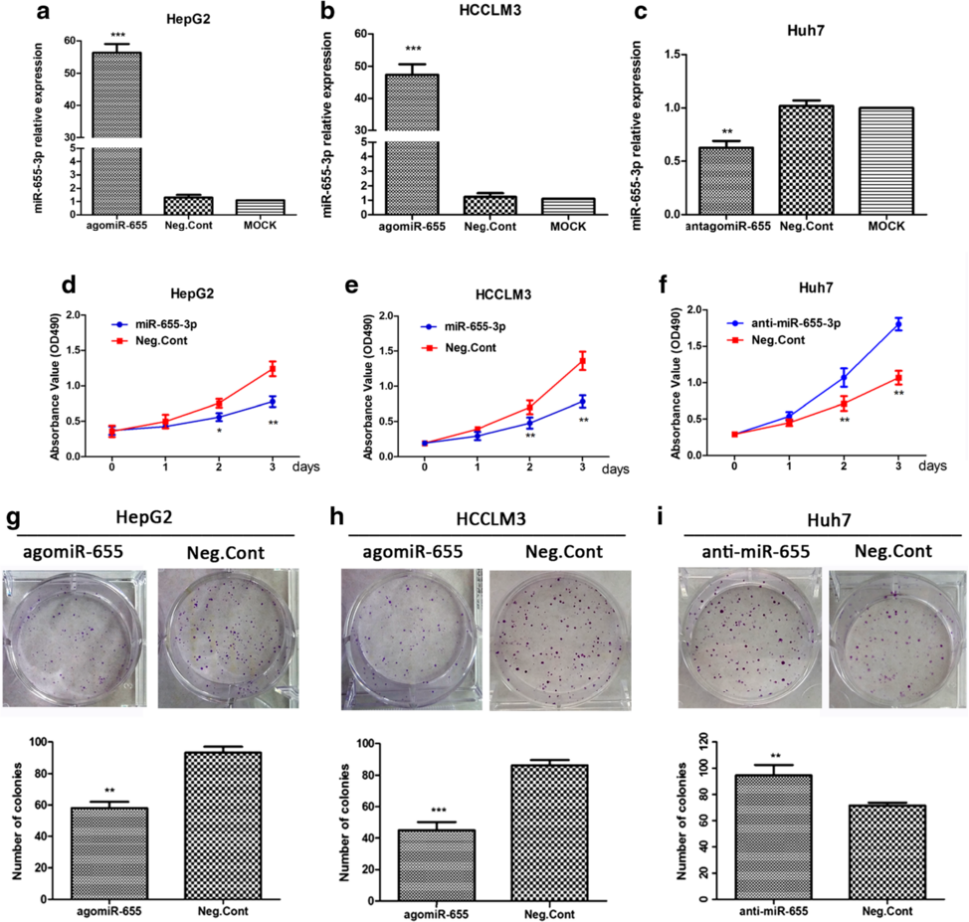
MiR-655-3p suppressed hepatocellular carcinoma cell growth and proliferation abilities. **a, b, c**. QRT-PCR analysis of miR-655-3p transfection efficiency after the miR-655-3p agomiR, or antagomiR transfection in HCC cells. **d, e, f**. The MTT assay analysis was used to evaluate the proliferation of HCC cells after transfection with the miR-655-3p agomiR, antagomiR or controls. **g, h, i**. Colony formation assay analysis of HCC cells after treatment with miR-655-3p agomiR or antagomiR or controls. (Data are mean ± SEM (*n* = 3), *p < 0.05, **p < 0.01, and ***p < 0.001 VS controls)

To examine the functional roles of miR-655-3p in HCC, we upregulated HCCLM3 and HepG2 cells by miR-655-3p agomiR (100nM) transfection. Overexpression of miR-655-3p in the two HCC cell lines were confirmed by qRT-PCR after transfection for 48 h (Fig. 2a, b). Then MTT and colony formation assays were performed to detected proliferation ability. Compared to the negative control group, the cancer cell proliferation was dramatically inhibited in miR-655-3p overexpression group by MTT analysis after transfection for 48 h and 72 h (Fig. 2d, e). Consistent with the MTT assay, colony formation assay also showed that miR-655-3p overexpression led to a significant reduction of colony number in HCC cells (Fig. 2g, h). Conversely, miR-655-3p inhibitor significantly promoted the proliferation potential in Huh7 cells both in MTT and colony formation assays (Fig. 2c, f, i). These results proved that miR-655-3p inhibit proliferation in HCC.

### Restoration of miR-655-3p represses migration and invasion of HCC cells

**Fig. 3:**
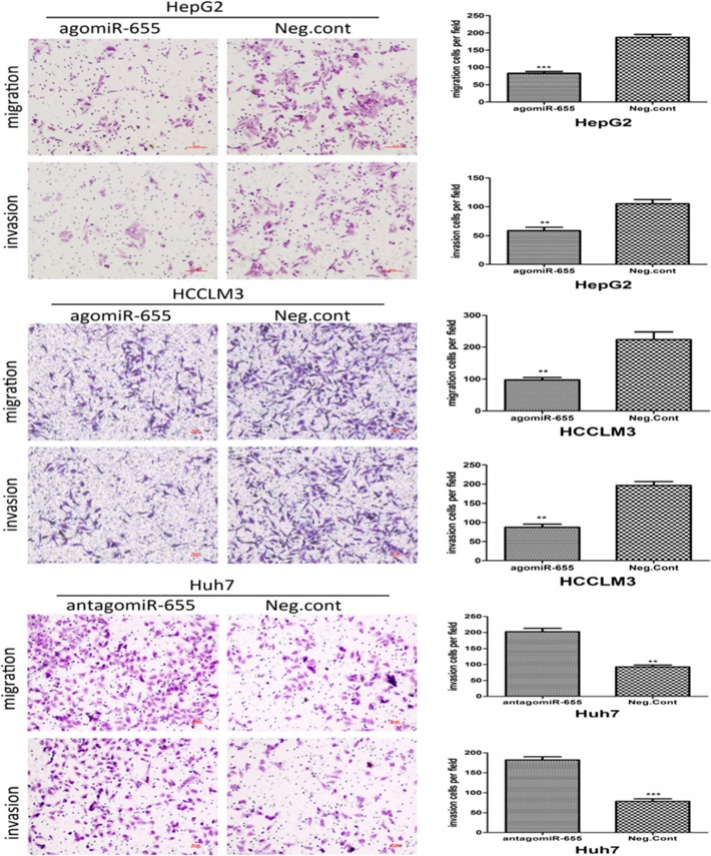
Effect of miR-655-3p on invasion and migration in HCC. Restoration of miR-655-3p repressed migration and invasion of HepG2 and HCCLM3 cells; and inhibiting miR-655-3p expression promoted cell migration and invasion in Huh7 cells. Transwell analysis was used. Data are mean ± SEM (*n* = 3). *p < 0.05, **p < 0.01, and ***p < 0.001 compared to controls

To investigate the function of miR-655-3p in cell migration and invasion, transwell chamber assay was performed in HCC cells. We found enhancement of the expression of miR-655-3p in HepG2 and HCCLM3 cells could significantly inhibit cell invasion and migration abilities. The number of invasive and migrated cells in the miR-655-3p overexpression group(82 ± 5 and 58 ± 6, respectively) was significantly decreased, compared with the negative control group (180 ± 8 and 105 ± 7, respectively) in HepG2 cells. The same results were also observed in HCCLM3 cells (97 ± 8 and 87 ± 8 vs. 212 ± 24 and 116 ± 10, respectively). Conversely, anti-agomiR-655-3p significantly increased the cell migration and invasion of the Huh7 cells (202 ± 10 and 182 ± 8 vs. 92 ± 6 and 79 ± 6) (Fig. 3). Based on these results, we concluded that miR-655-3p decreased the migration and invasion of HCC cells.

### MiR-655-3p can upregulate E-cadherin expression and inhibit β-catenin signal pathway in HCC cells

**Fig. 4:**
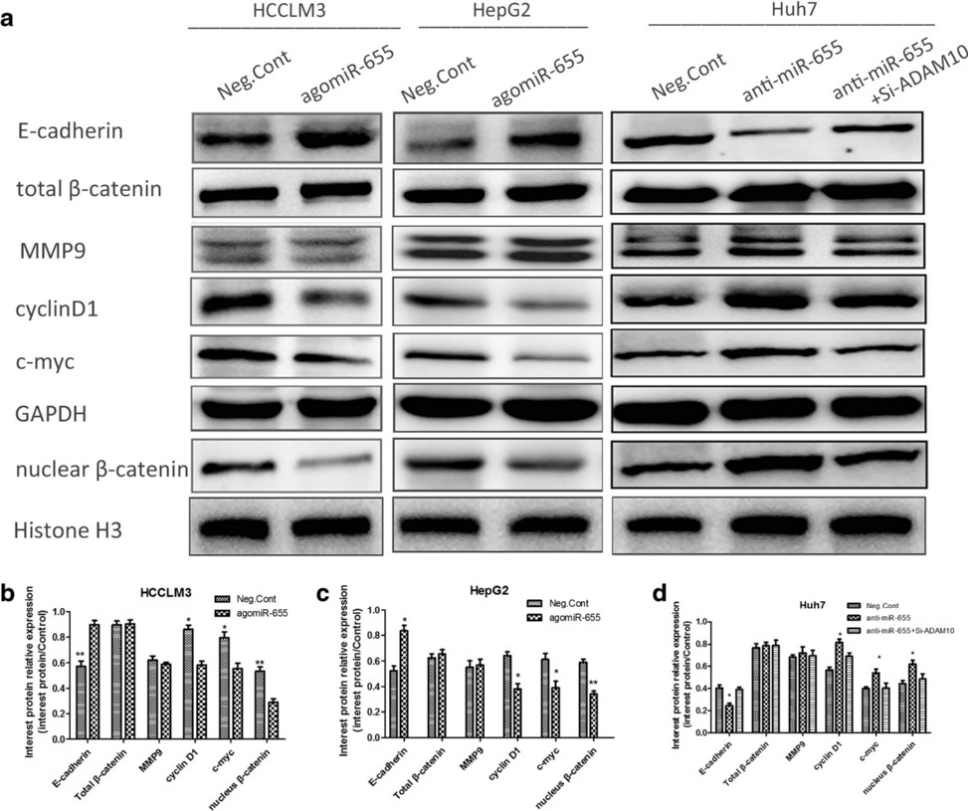
MiR-655-3p up-regulated E-cadherin expression and inhibited β-catenin signal pathway in HCC cells. **a** Western blot analysis of E-cadherin and β-catenin signal pathway proteins after transfection of miR-655-3p agomiR or antagomiR or controls. **b, c, d** Column charts were used to display the changes of protein expression. Gray value was analyzed by Image J Software. Histone H3 was used an internal control for nuclear β-catenin, and GAPDH for others. Data are mean ± SEM (*n* = 3). *p < 0.05, **p < 0.01, and ***p < 0.001 compared to controls

To better understand the underlying molecular mechanism of miR-655-3p inhibiting proliferation and metastasis of HCC, we detected the expression of potential proliferation and metastasis-associated proteins by western blot. The results showed that miR-655-3p up-regulation in HCCLM3 and HepG2 cells increased E-cadherin and decreased the nucleus β-catenin (the total β-catenin showed no change), cyclinD1, c-myc protein levels(Fig. 4a, b, c). Conversely, inhibiting miR-655-3p in Huh7 cells decreased E-cadherin and unregulated nucleus β-catenin, cyclinD1 and c-myc proteins (Fig. 4a, d). Only nucleus β-catenin decrease after miR-655-3p overexpression indicated that miR-655-3p may affect the distribution of β-catenin. All these results suggested that miR-655-3p might influence the biological behavior of HCC by regulating E-cadherin expression and inhibiting β-catenin signal pathway.

### ADAM10 is a direct target of miR-655-3p in HCC

It has been demonstrated miRNA relies on its regulating target genes to achieve its biological function [17]. Therefore, we identified miR-655 target genes using the target prediction tool, miRwalk, a comprehensive database on miRNAs with six established program (miRanda, miRDB, miRWalk, TargetScan, RNA22, and PITA) [18]. There were no miR-655-3p binding sites predicted on mRNA of E-cadherin, β-catenin, cyclinD1 and c-myc. While, ADAM10 was one of the gene that was predicted to binding miR-655 by at least five program and related to HCC biological progress according to the relevant previous reports [19, 20].

**Fig. 5:**
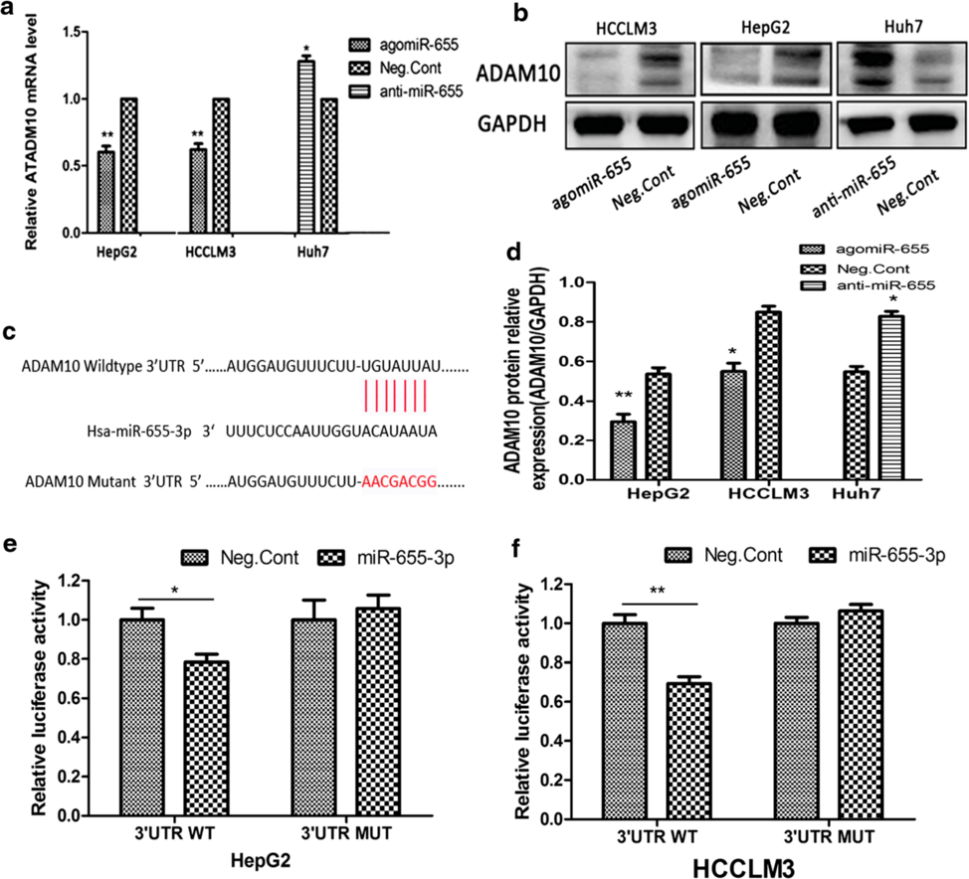
ADAM10 is a direct target of miR-655-3p in HCC. **a**. qRT-PCR analysis of ADAM10 mRNA expression after the miR-655-3p agomiR, or antagomiR transfection in HCC cells. **b, d**. Western blot analysis of ADAM10 protein expression after the miR-655-3p agomiR, or antagomiR transfection in HCC cells; and their Column charts analysis. **c**. The predicted interaction site of miR-655-3p and candidate target gene ADAM10 wild-type 3’-UTR and serial deleted forms of the 3’UTR reporters. **e, f**. Luciferase assay of co-transfected with miR-655-3p agomiR and pMIR-REPORT-ADAM10 plasmid (miR-NC and miR-655-3p with ADAM10 WT 3’UTR; miR-NC and miR-655-3p with ADAM10 MUT 3’UTR) after 24 h. Data are mean ± SEM (*n* = 3). *p < 0.05, and **p < 0.01 compared to controls

As predicted, overexpression of miR-655-3p in HepG2 and HCCLM3 cells decreased the expression of ADAM10 at both the mRNA and protein levels, whereas miR-655-3p inhibitor increased its expression in Huh7 cells (Fig. 5a, b, d). Using TargetScan, we located potential binding sites for miR-655-3p at the 3’UTR of ADAM10 mRNAs (Fig. 5c). Then, a dual-luciferase reporter system was carried out to determine whether ADAM10 was a direct target of miR-655-3p. Overexpression of miR-655-3p significantly suppressed the luciferase activity of the wild-type ADAM10 3’-UTR, but failed to affect the mutant 3’-UTR in HCCLM3 and HepG2 cells (Fig. 5e, f). Taken together, these results demonstrated that ADAM10 was a direct target of miR-655-3p.

### Loss-of function of ADAM10 mimicked impact of miR-655-3p on HCC cell proliferation and metastasis

**Fig. 6:**
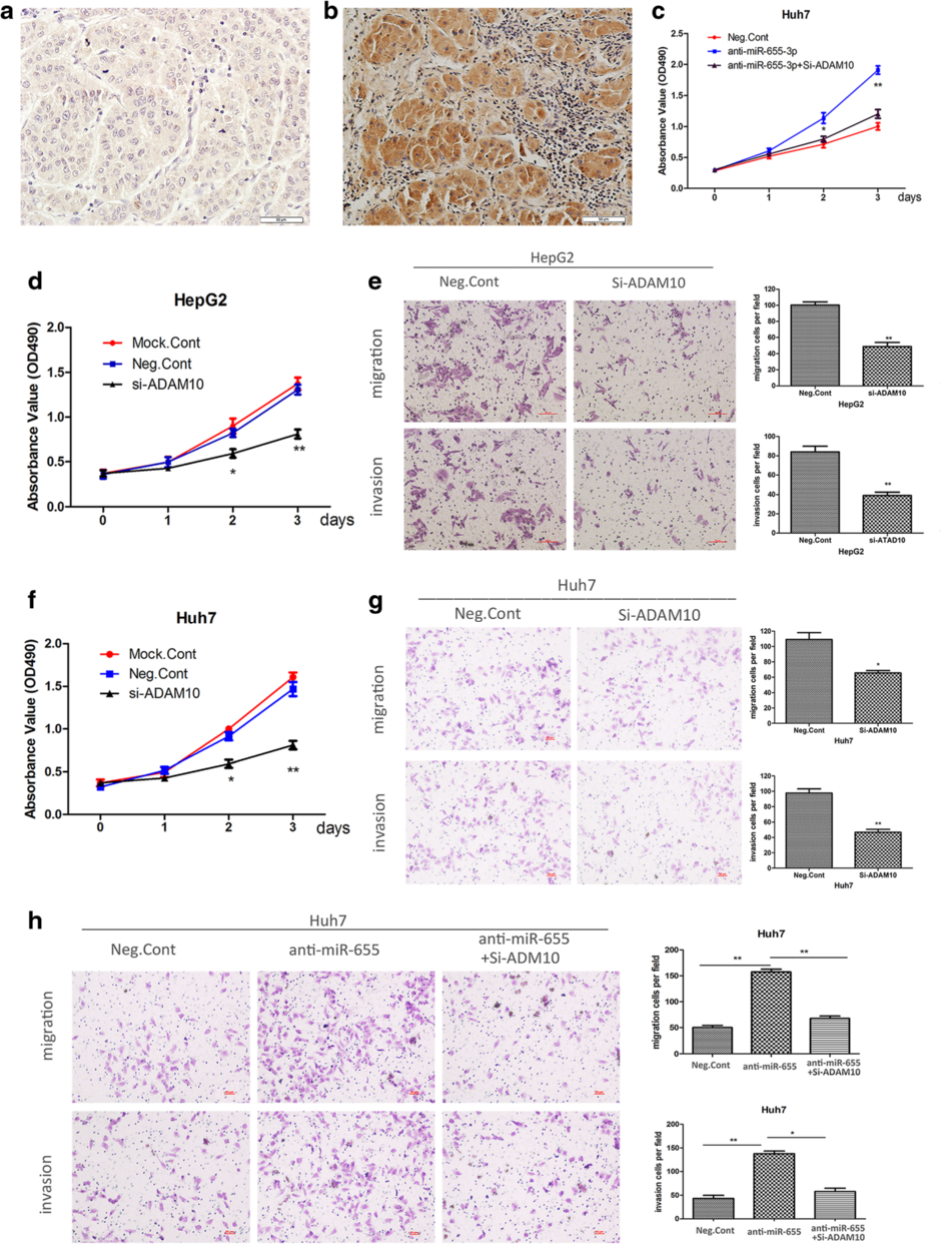
Loss-of function of ADAM10 mimicked impact of miR-655-3p on HCC cell proliferation and metastasis. **a, b** IHC staining of ADAM10 in HCC and normal liver tissues. **d, f** The MTT assay analysis of proliferation after Si-ADAM10 or controls transfected in HepG2 and Huh7 cells respectively. **e, g** Transwell analysis was used to detect the impact of migration and invasion after Si-ADAM10 or controls transfected in HepG2 and Huh7 cells respectively. **c** The MTT assay analysis of proliferation ability of Huh7 cells after transfected miR-655-3p antagomiR or co-transfected Si-ADAM10 or controls. **h** Transwell analysis of migration and invasion abilities of Huh7 cells after transfected miR-655-3p antagomiR or co-transfected Si-ADAM10 or controls. Data are mean ± SEM (*n* = 3). *p < 0.05, and **p < 0.01 compared to controls

To confirm whether miR-655-3p dependent repression of HCC cell biological behaviors was mediated by ADAM10, we investigated the expression and role of ADAM10 in HCC. ICH results showed that expression of ADAM10 is strongly positively stained in HCC tissues, while, absent or sporadic in non-tumorous liver tissues. And ADAM10 is located in the cytoplasm and cell membrane in HCC ADAM10-positive cells (Fig. 6a, b). The further results revealed that si-ADAM10 mediated downregulation of ADAM10 expression inhibited the proliferation, migration and invasion both in HepG2 and Huh7 cells (Fig. 6d, e, f, g). The effect of ADAM10 silencing was similar to the effect of miR-655-3p overexpression on proliferation, migration and invasion of HepG2 cells (Fig. 2d, Fig. 3). Moreover, inhibition of ADAM10 expression significantly attenuated the abilities of cell proliferation, migration and invasion promoted by anti-miR-655-3p in Huh7 cells (Fig. 6c, h). These results demonstrated that miR-655-3p inhibited the proliferation, migration and invasion of HCC cells by targeting ADAM10.

## Discussion

Tumor invasion, metastatic dissemination, recurrence, and drug resistance have been identified as the major causes of the poor clinical outcome in HCC patients [1–4]. More and more miRNAs were converged to account for characters of various tumor processes, including tumor initiation, development and metastasis [6, 21]. MiR-655-3p was encoded in 14q32 locus, a region that has been implicated in oncogenesis and metastasis of diverse cancer types [12, 13]. Yang et al. showed miR-655 expression was decreased in esophageal squamous cell carcinoma (ESCC) and overexpression of miR-655 inhibited ESCC cell invasiveness by targeting PTTG1 [14]. Yosuke et al. reported that miR-655 represses EMT progress through inducing inactivation of the TGF-β signaling pathway by targeting ZEB1 and TGFBR2, in ESCC [15]. Kitamura et al. demonstrated that miR-134/487b/655 cluster regulate TGF-β induced EMT and drug resistance to Gefitinib by targeting MAGI2 in lung adenocarcinoma cells [16]. In addation, Zhang et al. showed that Norcantharidin suppress glioblastoma cell invasion through modulation of miR-655-mediated SUMO-specific protease 6 translation [22]. Various publications have associated the dysregulation of miR-655 with cancer progress. However, the relationship between miR-655 and HCC remains unknown.

In this study, we first demonstrated that miR-655-3p was significantly down-regulated in human HCC tissues and cell lines. We also found that down-regulation of miR-655-3p expression levels was significantly associated with the tumor size, portal vein tumor thrombosis(PVTT) status, TNM stage and metastasis status. Using MTT, colony formation and transwell assays, we found that overexpression of miR-655-3p could suppress the proliferation, migration and invasion ability in HCC cells in vitro, indicating the crucial role of miR-655-3p in HCC development. Based on above results, miR-655-3p might was a tumor suppressor in HCC. Then, the underlying molecular mechanism of miR-655-3p inhibiting HCC progress was detected, and the results showed upregulating miR-655-3p expression increased E-cadherin protein but decreased the nucleus β-catenin, cyclinD1 and c-myc expression. β-catenin is known to interact with TCF/LEF family of transcription factors to induce gene expression, such as cyclinD1 and c-myc [21, 23–25]. These results indicate that miR-655-3p might influence the biological behavior of HCC by regulating E-cadherin expression and inhibiting β-catenin signal pathway.

A disintegrin and metalloproteinase 10 (ADAM10), has been proved to be upregulated in various cancers and involved in cancer progression and metastasis, such as pancreatic cancer, breast cancer, lung cancer and hepatocellular carcinoma [26–30]. ADAM10 is reported as a sheddase that can cleave transmembrane proteins such as amyloid precursor protein (APP), E-cadherin, N-cadherin, CD44 and Notch, all of which play a significant role in proliferation, migration, invasion or stemness of cancer cells [27, 28, 31–35]. Of particular importance is that ADAM10 is identified as an oncogene contributing to HCC progression, such as metastasis, invasion and drug resistance of Sorafenib [18, 19]. In our study, we also found that ADAM10 silencing suppresses HCC proliferation, migration and invasion in vitro, and using a luciferase-based reporter assay, we demonstrated that miR-655-3p could bind to a sequence within the 3’-UTR of ADAM10. MiR-655-3p-mediated control of ADAM10 expression was further validated by complementary gain and loss-of-function approaches. Thus, we conclude that miR-655-3p mediates HCC progress by targeting the 3’-UTR of ADAM10. Neha et al. reported that inhibiting ADAM10 expression subsequently diminishes β-catenin intracellular signaling and repress TCF/LEF target gene expression [27], which is similar to our founding that miR-655-3p could reduce E-cadherin protein level and inhibit β-catenin pathway by target regulating ADAM10 in HCC. Besides, ADAM10 can exert different functions regulated by multiple micrornas. For example, miR-144/451 inhibits cancer metastasis by targeting ADAMTS5 and ADAM10 in human epithelial cancers [36]; miR-122-5p reduces trastuzumab resistance by regulating ADAM10 in breast cancer [37]; miR-140-5p can repress tumor progression by targeting ADAM10 in human tongue and hypopharyngeal squamous cancer cells [38, 39]. Our study provided a new mechanism accounting for ADAM10 dysregulation in HCC.

## Conclusion

In conclusion, our study demonstrate that miR-655-3p functions as tumor suppressor by directly targeting ADAM10 and indirectly regulating β-catenin pathway in HCC progression and metastasis. These findings provide a new insight into the molecular pathogenesis of HCC and identify miR-655-3p as a novel therapeutic candidate target for HCC.

## Abbreviations

3’-UTR, 3’ untranslated region; ADAM10, A Disintegrin and Metalloprotease Domain; EMT, epithelial mesenchymal transition; ESCC, esophageal squamous cell carcinoma; HCC, Hepatocellular carcinoma; miRNAs, microRNAs; MTT, 3-(4,5)-dimethylthiahiazo(-z-y1)-3,5-di-phenytetrazoliumromide; PVTT, portal vein tumor thrombosis; qRT-PCR, real time polymerase chain reaction; TCF/LEF, T-cell factor and Lymphoid enhancing factor.
